# Early COVID‐19 XBB.1.5 Vaccine Effectiveness Against Hospitalisation Among Adults Targeted for Vaccination, VEBIS Hospital Network, Europe, October 2023–January 2024

**DOI:** 10.1111/irv.13360

**Published:** 2024-08-15

**Authors:** Liliana Antunes, Clara Mazagatos, Iván Martínez‐Baz, Reinout Naesens, Maria‐Louise Borg, Goranka Petrović, Terra Fatukasi, Ligita Jancoriene, Ausenda Machado, Beatrix Oroszi, Petr Husa, Mihaela Lazar, Ralf Dürrwald, Jennifer Howard, Aryse Melo, Gloria Pérez‐Gimeno, Jesús Castilla, Eva Bernaert, Aušra Džiugytė, Zvjezdana Lovrić Makarić, Margaret Fitzgerald, Auksė Mickienė, Verónica Gomez, Gergő Túri, Lenka Součková, Alexandru Marin, Kristin Tolksdorf, Nathalie Nicolay, Angela M. C. Rose, the European Hospital Vaccine Effectiveness Group

**Affiliations:** ^1^ Epidemiology Department Epiconcept Paris France; ^2^ National Centre for Epidemiology Institute of Health Carlos III Madrid Spain; ^3^ Consortium for Biomedical Research in Epidemiology and Public Health (CIBERESP) Madrid Spain; ^4^ Instituto de Salud Pública de Navarra – IdiSNA Pamplona Spain; ^5^ Department of Medical Microbiology and Infection Prevention & Control Ziekenhuisnetwerk Antwerpen Antwerp Belgium; ^6^ Infectious Disease Prevention and Control Unit (IDCU) Health Promotion and Disease Prevention Msida Malta; ^7^ Croatian Institute of Public Health Zagreb Croatia; ^8^ Health Service Executive‐Health Protection Surveillance Centre (HPSC) Dublin Ireland; ^9^ Clinic of Infectious Diseases and Dermatovenerology, Institute of Clinical Medicine, Medical Faculty Vilnius University Vilnius Lithuania; ^10^ Epidemiology Department National Health Institute Doutor Ricardo Jorge Lisbon Portugal; ^11^ National Laboratory for Health Security, Epidemiology and Surveillance Centre Semmelweis University Budapest Hungary; ^12^ University Hospital Brno Masaryk University Brno Czechia; ^13^ Cantacuzino National Military‐Medical Institute for Research and Development Bucharest Romania; ^14^ National Reference Centre for Influenza Robert Koch Institute Berlin Germany; ^15^ Infectious Diseases Department National Health Institute Doutor Ricardo Jorge Lisbon Portugal; ^16^ Department of Infectious Diseases Lithuanian University of Health Sciences Kaunas Lithuania; ^17^ Dr Victor Babes Clinical Hospital of Infectious and Tropical Diseases Bucharest Romania; ^18^ Department for Infectious Disease Epidemiology Robert Koch Institute Berlin Germany; ^19^ European Centre for Disease Prevention and Control Stockholm Sweden

**Keywords:** COVID‐19 XBB.1.5 vaccine, Europe, SARS‐CoV‐2 hospitalisation, vaccine effectiveness

## Abstract

We conducted a multicentre test‐negative case–control study covering the period from October 2023 to January 2024 among adult patients aged ≥ 18 years hospitalised with severe acute respiratory infection in Europe. We provide early estimates of the effectiveness of the newly adapted XBB.1.5 COVID‐19 vaccines against PCR‐confirmed SARS‐CoV‐2 hospitalisation. Vaccine effectiveness was 49% overall, ranging between 69% at 14–29 days and 40% at 60–105 days post vaccination. The adapted XBB.1.5 COVID‐19 vaccines conferred protection against COVID‐19 hospitalisation in the first 3.5 months post vaccination, with VE > 70% in older adults (≥ 65 years) up to 1 month post vaccination.

## 1 Introduction

The European Medicines Agency (EMA) authorised three adapted XBB.1.5 COVID‐19 vaccines between August and October 2023: Comirnaty Omicron XBB.1.5, Spikevax XBB.1.5 and Nuvaxovid XBB.1.5 [1]. In the European Union/European Economic Area (EU/EEA), between 1 September 2023 and 15 January 2024, 99% of all vaccines administered were adapted XBB.1.5, with Comirnaty Omicron XBB.1.5 accounting for 97% of all products [2]. The XBB.1.5‐like+F456L variant was dominating in the EU/EEA from autumn 2023 to mid‐December 2023, while the BA.2.86 SARS‐CoV‐2 variant started to dominate in European Union/European Economic Area (EU/EAA) countries by 18 December 2023 [3]. We aimed to estimate early vaccine effectiveness (VE) of the newly adapted XBB.1.5 COVID‐19 vaccines against PCR‐confirmed SARS‐CoV‐2 hospitalisation in Europe.

## 2 VEBIS Hospital Network

**FIGURE 1:**
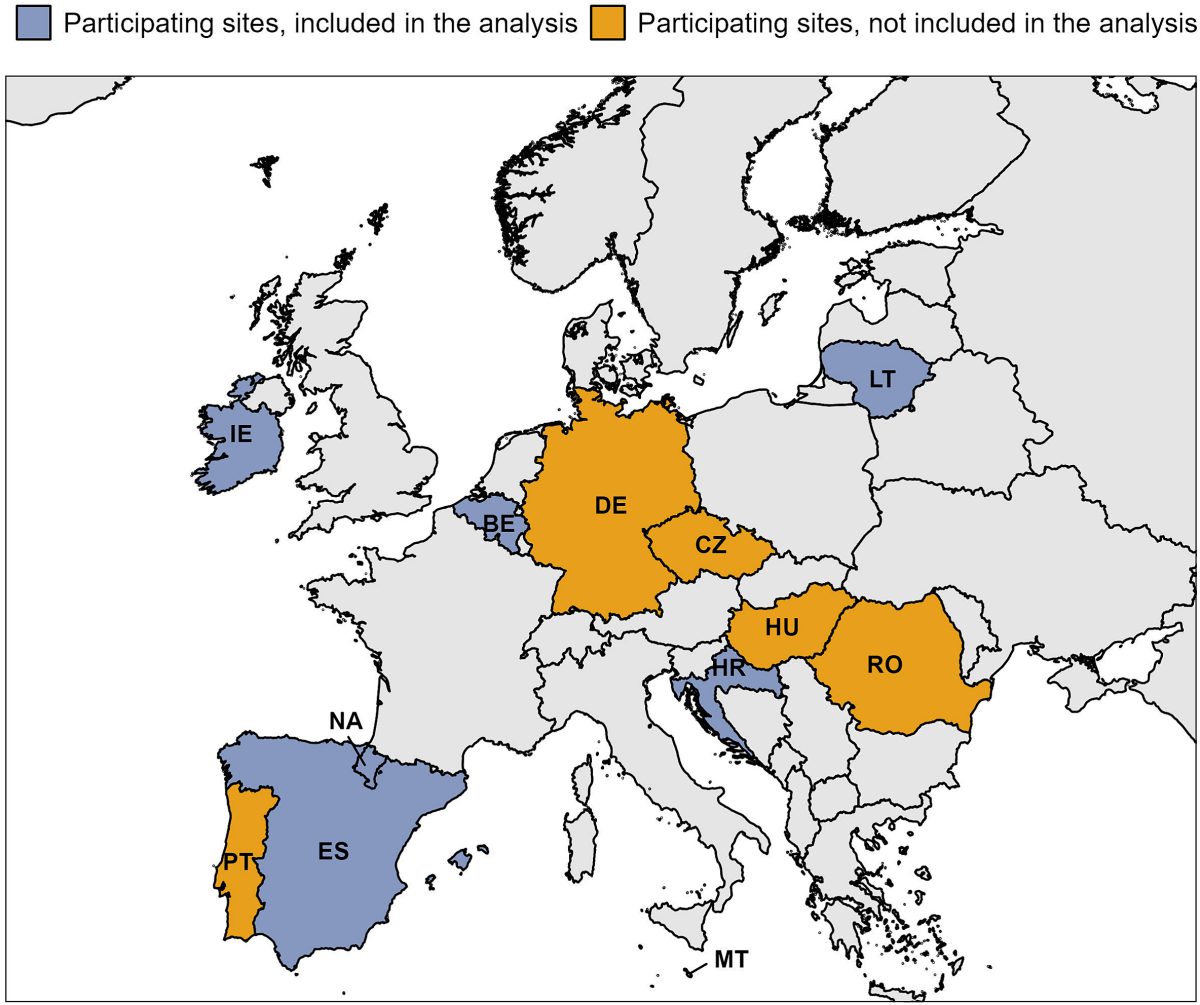
Countries and study sites included in the VEBIS hospital network, Europe, 05 October 2023–14 January 2024.VEBIS: Vaccine Effectiveness, Burden and Impact Studies Twelve participating sites: Belgium (BE), Croatia (HR), Czechia (CZ), Germany (DE), Hungary (HU), Ireland (IE), Lithuania (LT), Malta (MT), Navarre region, Spain (NA), Portugal (PT), Romania (RO) and Spain (ES). Included in this analysis: BE, HR, IE, LT, MT, NA and ES.

The Vaccine Effectiveness, Burden and Impact Studies (VEBIS) hospital study is a multicentre, test‐negative, case–control study. The VEBIS hospital network includes 65 hospitals in 11 participating EU/EEA countries (Figure 1), following a common generic protocol [4]. We included patients with severe acute respiratory infection (SARI), defined as those hospitalised for ≥ 24 h, with at least one of the following symptoms: fever, cough, shortness of breath or sudden onset of anosmia, ageusia or dysgeusia [5], adapted for local use. We defined cases as SARI patients testing positive for SARS‐CoV‐2 by PCR within 48 h of admission or, if known, in the previous 14 days and controls as PCR‐negative within 48 h, with no positive test in the previous 14 days (if known). We excluded SARI patients with missing or erroneous information on key variables (sex, age, chronic conditions and dates of onset, swab and hospital admission), as well as sites with fewer than five cases or controls or with no vaccinated patients in both case and control groups (Figure S1). We restricted the analysis to adults ≥ 18 years, targeted by the vaccine recommendations in each study site (older adults aged ≥ 50, ≥ 55, ≥ 60 or ≥ 65 years old and those in medical risk groups, as defined locally; Table S2). The study period began 14 days after the start of the 2023 autumn vaccination campaign, varying by study site (Table S1). Overall, the study period was from 05 October 2023 to 14 January 2024.

## 3 Vaccination Definition and SARI Patient Description

We defined SARI patients as vaccinated if they received their most recent COVID‐19 vaccine dose after the start of the 2023 autumn vaccination campaign in their study site, regardless of their previous vaccination history, and at least 14 days before symptom onset. Where known, we excluded patients with a vaccine other than XBB.1.5 or with vaccine brand other than Comirnaty, Spikevax or Nuvaxovid (information not collected by all sites). We defined unvaccinated SARI patients as those who had never received a COVID‐19 vaccine or with last dose received at least 180 days prior to the start of the 2023 autumn vaccination campaign in each study site. We excluded those vaccinated 1–13 days before symptom onset.

We included 622 SARS‐CoV‐2 cases and 3457 test‐negative controls aged ≥ 18 years from 41 hospitals in seven sites, after exclusions (one site was excluded for data quality issues, one had fewer than five cases and three had no vaccinated patients in both case and control groups; Figure S1).

A total of 171 (27%) cases and 1513 (44%) controls had been vaccinated during the site‐specific autumn vaccination campaign. The median time between vaccination and symptom onset was 58 days (interquartile range [IQR] 42–71) for cases and 52 days (IQR 35–67) for controls (Table 1).

**TABLE 1:** Characteristics of the SARI patients by case and control status, VEBIS hospital network, Europe, 05 October 2023–14 January 2024 (*n* = 4079).

Characteristic	SARS‐CoV‐2 cases (*n* = 622)		Test‐negative controls (*n* = 3457)		
	*n*	%	*n*	%	
Age (years)					
18–59	58	9	390	11	
60–79	264	42	1610	47	
≥ 80	300	48	1457	42	
≥ 65	526	85	2802	81	
Median (IQR)	79 (71–86)		77 (67–85)		
Male	328	53	1758	51	
Any chronic condition^a^	514	83	2888	84	
Vaccination status at time of symptom onset					
Vaccinated during 2023 autumn campaign^b^	171	27	1513	44	
Not vaccinated during 2023 autumn campaign^c^	451	73	1944	56	
Days since last dose received during 2023 autumn vaccination campaign at time of symptom onset^d^					
14–29 days	18	11	277	18	
30–59 days	75	44	680	45	
≥ 60 days	78	46	556	37	
Median (IQR)	58 (42–71)		52 (35–67)		

Abbreviations: SARI: severe acute respiratory infection; VEBIS: Vaccine Effectiveness, Burden and Impact Studies.

^a^ Common chronic conditions: diabetes, heart disease, lung disease, asthma and immunodeficiency.

^b^ Vaccinated: received a COVID‐19 vaccine dose after the start of the 2023 autumn vaccination campaign in each study site. Start dates for the 2023 autumn vaccination campaign are shown in Table S1.

^c^ Not vaccinated: never vaccinated for COVID‐19 or with last COVID‐19 vaccination dose received 180 days prior to the start of the vaccination campaign in each study site. Start dates of the seasonal 2023/24 vaccination campaign are in Table S1.

^d^ Restricted to those vaccinated during the 2023 autumn vaccination campaign.

## 4 Vaccine Effectiveness

We estimated the odds ratio (OR) of vaccination between cases and controls using logistic regression with study site as a fixed effect and adjusted for date of symptom onset, sex, age and presence of a chronic condition. The VE was calculated as (1 − OR) × 100%. We estimated VE overall and by time since vaccination (TSV) in 30‐day bands, for all adults and by age group (18–59, 60–79, ≥ 80 and ≥ 65 years). Estimates were not shown if there were < 20 vaccinated patients; fewer than five vaccinated/unvaccinated cases or controls; or when the OR estimate had an absolute difference > 10% from the OR estimated using penalised logistic regression (to assess small sample bias).

We conducted the following sensitivity analyses: (1) inclusion of SARI patients with symptom onset at least 7 days (instead of 14 days) after vaccination; (2) VE for those with symptom onset in the period 7–13 days post vaccination.

The VE for all individuals aged ≥ 18 years was 49% [95%CI: 37;58] overall; 69% [95%CI: 50;82] in the first 14–29 days post vaccination, 46% [95%CI: 29;59] between 30 and 59 days; and 40% [95%CI: 19;55] between 60 and 105 days (Table 2).

**TABLE 2:** Vaccine effectiveness of the adapted COVID‐19 XBB.1.5 vaccines against hospitalisation, overall and by time since vaccination, for individuals ≥ 18 years old in the target group for vaccination^a^ and by age group, VEBIS hospital network, Europe, 05 October 2023–14 January 2024 (*n* = 4079).

Individuals from the target group for vaccination	Vaccination status/time since vaccination	SARS‐CoV‐2 cases	Test‐negative controls	Median (IQR) time since last dose to symptom onset^b^	Vaccine effectiveness (%)^c^	95% CI
All individuals^d^	Unvaccinated^e^	451	1944	643 (399–744)	Ref	—
	Vaccinated^f^	171	1513	53 (35–67)	49	37;58
	14–29 days	18	277	22 (18–26)	69	50;82
	30–59 days	75	680	46 (38–53)	46	29;59
	60–105 days	78	556	71 (65–76)	40	19;55
18–59 years	Unvaccinated^e^	53	327	698 (636–770)	Ref	—
	Vaccinated^f^	5	63	47 (34–63)	49	−28;84
	14–29 days	1	11	24 (19–25)	—	—
	30–59 days	4	32	44 (36–51)	—	—
	60–105 days	0	20	67 (63–75)	—	—
60–79 years	Unvaccinated^e^	198	956	664 (402–741)	Ref	—
	Vaccinated^f^	66	654	51 (34–63)	44	23;60
	14–29 days	9	127	22 (18–26)	59	20;81
	30–59 days	30	314	46 (38–53)	42	13;63
	60–105 days	27	213	68 (63–76)	36	−2;61
≥ 80 years^g^	Unvaccinated^e^	200	661	432 (385–745)	Ref	—
	Vaccinated^f^	100	796	55 (37–70)	52	36;64
	14–29 days	8	139	22 (18–25)	76	53;90
	30–59 days	41	334	47 (39–54)	55	34;70
	60–105 days	51	323	72 (67–77)	39	10;59
≥ 65 years^g^	Unvaccinated^e^	367	1421	447 (391–742)	Ref	—
	Vaccinated^f^	159	1381	53 (35–68)	50	38;60
	14–29 days	15	252	22 (18–26)	72	54;85
	30–59 days	68	610	46 (38–53)	48	31;62
	60–105 days	76	519	71 (66–77)	38	16;55

Abbreviations: CI: confidence interval; IQR: interquartile range; SARI: severe acute respiratory infection; VEBIS: Vaccine Effectiveness, Burden and Impact Studies.

^a^ Groups targeted for the 2023 autumn COVID‐19 vaccination include older adults (aged ≥ 50, ≥ 55, ≥ 60 or ≥ 65 years old) and those in medical risk groups, as defined locally by each study site (Table S2).

^b^ Only among patients that have received at least one COVID‐19 vaccine dose. The interquartile range (IQR) is presented as the first (25% percentile) and third (75% percentile) quartile of the time since vaccination among patients that have received at least one COVID‐19 vaccine dose.

^c^ The odds ratio (OR) of vaccination is estimated using logistic regression model with fixed effects, adjusted for site, date of symptom onset, sex, age and presence of any chronic condition (diabetes, heart disease, lung disease, asthma and immunodeficiency). The best functional forms of the continuous variables age and onset date (categories, splines, linear terms) were selected using the Akaike information criterion. Vaccine effectiveness is given by VE = (1 − OR) × 100%.

^d^ All individuals: all those in the target group for vaccination in their study site and aged at least 18 years old.

^e^ Unvaccinated: SARI patients who did not receive a vaccine during the 2023 autumn vaccination campaign and were either never vaccinated for COVID‐19 or received their last COVID‐19 vaccination dose at least 180 days prior to the start of the 2023 autumn vaccination campaign in their study site.

^f^ Vaccinated: SARI patients who received a COVID‐19 vaccine dose after the start of the 2023 autumn vaccination campaign in their study site, at least 14 days before symptom onset. Patients vaccinated 1–13 days before symptom onset were excluded. We excluded patients with a vaccine other than XBB.1.5 or vaccine brand other than Comirnaty, Spikevax or Nuvaxovid, for the patients with known brand and/or type of the most recent vaccine dose.

^g^ Maximum age included in the analysis: 105 years.

For those aged 18–59 years old, VE was 49% [95%CI: −28;84] overall (Table 2). Small sample size precluded VE estimates by TSV in this age group. Among those 60–79 years old, VE was 44% [95%CI: 23;60] overall, 59% [95%CI: 20;81] in the first 29 days, 42% [95%CI: 13;63] between 30 and 59 days and 36% [95%CI: −2;61] after 60 days (Table 2). For individuals aged ≥ 80 years, VE was 52% [95%CI: 36;64] overall, 76% [95%CI: 53;90] in the first 29 days, 55% [95%CI: 34;70] between 30 and 59 days and 39% [95%CI: 10;59] after 60 days (Table 2).

The VE observed for those aged ≥ 65 years was similar to VE for those aged ≥ 18 years in the target group for vaccination (Table 2).

Sensitivity analyses including patients with symptom onset from 7–29 instead of 14–29 days post vaccination showed mostly lower VE, with absolute differences ranging between 0% and 13%. The VE for those with onset 7–13 days after vaccination ranged between 20% and 28% across groups, albeit with very low precision.

## 5 Discussion

We estimated VE of 49% against SARS‐CoV‐2 hospitalisation for the adapted XBB.1.5 vaccine in the period 05 October 2023–14 January 2024 in Europe. Results by age group showed slightly higher VE point estimates for those aged ≥ 80 years compared to those aged 60–79 years, with overlapping confidence intervals for all estimates. VE declined with TSV, from 69% in the first 29 days to 40% at 60–105 days.

Decline in VE over time has been reported in the literature for other COVID‐19 vaccines [6, 7, 8]. The decline might be explained by waning immunity or the SARS‐CoV‐2 variants in circulation. During our study period, XBB lineages (mostly XBB.1.5‐like+F456L) dominated in EU/EEA countries until 18 December 2023, when the BA.2.86 variant became predominant [3]. A small number of samples from cases included in the study was sequenced from one study site (35; 5.6%). Of these, 80% were identified as XBB and 20% as BA.2.86.

A study using the screening method in the Netherlands estimated VE against hospitalisation among ≥ 60 years between October and December 2023 to be 70.7% [9]. A cohort study in Denmark, during October 2023, found vaccination to be associated with a 76% reduced risk of COVID‐19 hospitalisation [10]. A preprint of a test‐negative study in United States among ≥ 18 years, between 11 October and 10 December 2023, estimated a similar VE against hospital admission to be 63% [11]. Our results are slightly lower, possibly explained by our longer median TSV. The US VISION network reported VE among patients aged ≥ 18 years to be 53% in the first 7–59 days post vaccination and 50% at 60–119 days [12]. Among patients aged ≥ 65 years, VE was 54% in the first 7–59 days and 50% at 60–119 days. The IVY network estimated VE of 43% among adults aged ≥ 18 years and 48% among adults aged ≥ 65 years. Our results are consistent with these, albeit with a greater decline in VE with increasing TSV, which might be explained by the different choice of TSV bands. Three of these studies included patients as early as 7 days post vaccination [9, 10, 12], whereas our vaccination eligibility cut‐off was at least 14 days.

Our study had some limitations. Although cases are required to meet the SARI case definition and to have a positive SARS‐CoV‐2 PCR result, they might have been hospitalised for reasons other than COVID‐19, which might underestimate our VE [13]. In addition, we assumed that vaccines received during the 2023 autumn vaccination campaign were all adapted XBB.1.5 COVID‐19 vaccines. We excluded non‐XBB.1.5 COVID‐19 vaccines where known, but since vaccine type and brand are not collected by all sites, we were unable to confirm this for all patients. However, it has been reported that 99% of all COVID‐19 vaccines administered in EU/EEA from 1 September 2023 to 15 January 2024 were adapted XBB.1.5 vaccines (either Pfizer BioNTech – Comirnaty Omicron XBB.1.5, Moderna – Spikevax XBB.1.5 or Novavax – Nuvaxovid XBB.1.5) [2]. Due to lack of information from all sites regarding previous COVID‐19 doses received, we could not exclude vaccinated patients who had received a previous COVID‐19 vaccine within 180 days prior to the start of the vaccination campaign (as was done for the unvaccinated). Finally, a small sample size for those aged 18–59 years resulted in low precision, and VE by TSV could not be estimated for this group.

Our multicentre study also had many strengths. We were able to include data from several countries and sites, allowing us a larger sample size and increased representativeness of the European population, for a pooled VE estimate that might be more generalisable. In addition, sites participating in the network follow a generic protocol, which helps to mitigate potential sources of heterogeneity, and increase internal validity of VE estimates.

The findings of our study suggest that the adapted COVID‐19 XBB.1.5 vaccines provided protection against hospitalisation in the first 3.5 months post vaccination, by reducing the risk of COVID‐19 hospitalisation by approximately half among the vaccinated individuals.

## Author Contributions

**Liliana Antunes:** conceptualization, methodology, writing – original draft, writing–review and editing, visualization, validation, software, formal analysis, data curation. **Clara Mazagatos:** investigation, methodology, writing – review and editing, data curation, resources, supervision. **Iván Martínez‐Baz:** investigation, methodology, writing – review and editing, data curation, resources, supervision. **Reinout Naesens:** investigation, methodology, writing – review and editing, data curation, resources, supervision. **Maria‐Louise Borg:** investigation, methodology, writing – review and editing, resources, data curation, supervision. **Goranka Petrović:** investigation, methodology, writing – review and editing, data curation, resources, supervision. **Terra Fatukasi:** investigation, methodology, writing – review and editing, data curation, resources, supervision. **Ligita Jancoriene:** investigation, methodology, writing – review and editing, data curation, supervision, resources. **Ausenda Machado:** investigation, methodology, writing–review and editing, data curation, supervision, resources. **Beatrix Oroszi:** investigation, methodology, writing – review and editing, data curation, supervision, resources. **Petr Husa:** investigation, methodology, writing – review and editing, data curation, supervision, resources. **Mihaela Lazar:** investigation, methodology, writing – review and editing, supervision, resources, data curation. **Ralf Dürrwald:** investigation, methodology, writing – review and editing, data curation, supervision, resources. **Jennifer Howard:** methodology, writing – review and editing, data curation, software, visualization. **Aryse Melo:** investigation, writing – review and editing, data curation, resources, visualization, supervision, methodology. **Gloria Pérez‐Gimeno:** investigation, methodology, writing – review and editing, data curation, supervision, resources. **Jesús Castilla:** investigation, methodology, writing – review and editing, data curation, supervision, resources. **Eva Bernaert:** investigation, methodology, writing – review and editing, data curation, supervision, resources. **Aušra Džiugytė:** investigation, methodology, writing – review and editing, data curation, supervision, resources. **Zvjezdana Lovrić Makarić:** investigation, methodology, writing – review and editing, data curation, supervision, resources. **Margaret Fitzgerald:** investigation, methodology, writing – review and editing, data curation, supervision, resources. **Auksė Mickienė:** investigation, methodology, writing – review and editing, supervision, resources, data curation. **Verónica Gomez:** investigation, methodology, writing – review and editing, data curation, supervision, resources. **Gergő Túri:** investigation, methodology, writing – review and editing, data curation, resources. **Lenka Součková:** investigation, methodology, writing – review and editing, data curation, resources. **Alexandru Marin:** investigation, methodology, writing – review and editing, data curation, resources. **Kristin Tolksdorf:** investigation, methodology, writing – review and editing, data curation, resources, supervision. **Nathalie Nicolay:** conceptualization, project administration, writing – review and editing, validation. **Angela M. C. Rose:** conceptualization, funding acquisition, supervision, project administration, methodology, writing – review and editing, validation. **on behalf of the European Hospital Vaccine Effectiveness Group:** conceptualization, methodology, data curation, resources, writing – review and editing, investigation.

## Ethics Statement

The planning, conduct and reporting of the studies was in line with the Declaration of Helsinki. Official ethical approval was not required if studies were classified as being part of routine care/surveillance (Spain, Ireland, Malta); for the Netherlands, the study was not subject to the Dutch Medical Research with Human Subjects Law (Wet Medisch‐wetenschappelijk onderzoek met mensen, WMO) as it is non‐interventional and uses routine clinical data only and data were collected retrospectively. In Belgium and Germany, VE estimation is included in SARI surveillance. For Belgium, the protocol was approved by the central Ethical Committee (CHU ST Pierre, Bruxelles) and each participating hospital's local ethical committees in 2011 (AK/12‐02‐11/4111), updated in 2014 (B.U.N. 143201215671). The German SARI surveillance was approved by the Charité‐Universitätsmedizin Berlin Ethical Board (Reference EA2/218/19). Other study sites obtained local ethical approval from a national review board (Croatia: approved 24 May 2021 and 26 January 2022, Ethics Committee of the Croatian Institute of Public Health, Klasa: 030‐02/21‐01/1, Ur.broj: 381‐15‐21‐7; Klasa: 030‐02/21‐01/1, Ur.broj: 381‐15‐22‐14; France: eighth amendment approved 28 May 2021 by the French Data Protection Agency, and the French ethics research committee ‘Comité de Protection des Personnes’; Hungary: approved in March 2021 by the National Scientific and Ethical Committee for the period 01 September 2021–01 September 2024 [IV/1885‐5/2021/EKU]; Lithuania: approved 11 May 2021 by Lithuanian Biomedical Research Ethics Committee, No. 6B‐21‐85; Navarra: PI2020/45; Portugal: approved 19 January 2021 by the Ethics Committee of Instituto Nacional de Saúde Doutor Ricardo Jorge, no registration number given; Romania: approved by the Ethics Committee of the Ministerul Apărării Naţionale Institutul Naţionale de Cercetare pentru Dezvoltare Medico‐Militară ‘Cantacuzino’ for the period 2022–2023, No. CE199/2022).

## Conflicts of Interest

Aukse Mickiene has received a grant for the Independent Investigator Initiated Research (Project Code/PO/Tracking Number WI236259; Grant ID#53233947); Pfizer R&D Investigator‐Initiated Research program (https://www.pfizer.com/science/collaboration/investigator‐initiated‐research) for the scientific project ‘A prospective study on the long‐term outcome and pathogenesis of tick‐borne encephalitis’ and a Grant from the European Society of Clinical Microbiology and Infectious Diseases (ESCMID) Study Group for Infectious Diseases of the Brain (ESGIB); sponsorship for participation in the international scientific conferences by MSD, Pfizer, Abbvie, Janssen, payments for lectures in local scientific conferences and consultation fees from GSK, Sanofi, Pfizer and E‐visit.

Ligita Jancoriene has received honoraria fees for lectures from Pfizer, Viatris and Swixx Biopharma.

All other authors declare no conflicts of interest.

## Supporting information


**Figure S1** Patient exclusion flowchart, VEBIS hospital study, October 2023–January 2024.
**Table S1.** Start date of the 2023 autumn vaccination campaign by site, VEBIS hospital study, October 2023–January 2024.
**Table S2.** Target groups for vaccination during the 2023 COVID‐19 autumn vaccination campaign by site^a^, VEBIS hospital study, October 2023–January 2024.

## Data Availability

Data are available on request.
