# Supplementary material for: Early COVID‐19 XBB.1.5 Vaccine Effectiveness Against Hospitalisation Among Adults Targeted for Vaccination, VEBIS Hospital Network, Europe, October 2023–January 2024
Source: Influenza Other Respir Viruses. 2024 Aug 15;18(8):e13360. doi: 10.1111/irv.13360 (PMC11325250; doi:10.1111/irv.13360)
Supplement: Supplementary file 1 — Figure S1 Patient exclusion flowchart, VEBIS hospital study, October 2023–January 2024. Table S1. Start date of the 2023 autumn vaccination campaign by site, VEBIS hospital study, October 2023–January 2024. Table S2. Target groups for vaccination during the 2023 COVID‐19 autumn vaccination campaign by sitea, VEBIS hospital study, October 2023–January 2024. [file IRV-18-e13360-s001.docx]

## Supplementary Figure S1. Patient exclusion flowchart, VEBIS hospital study, October 2023 - January 2024


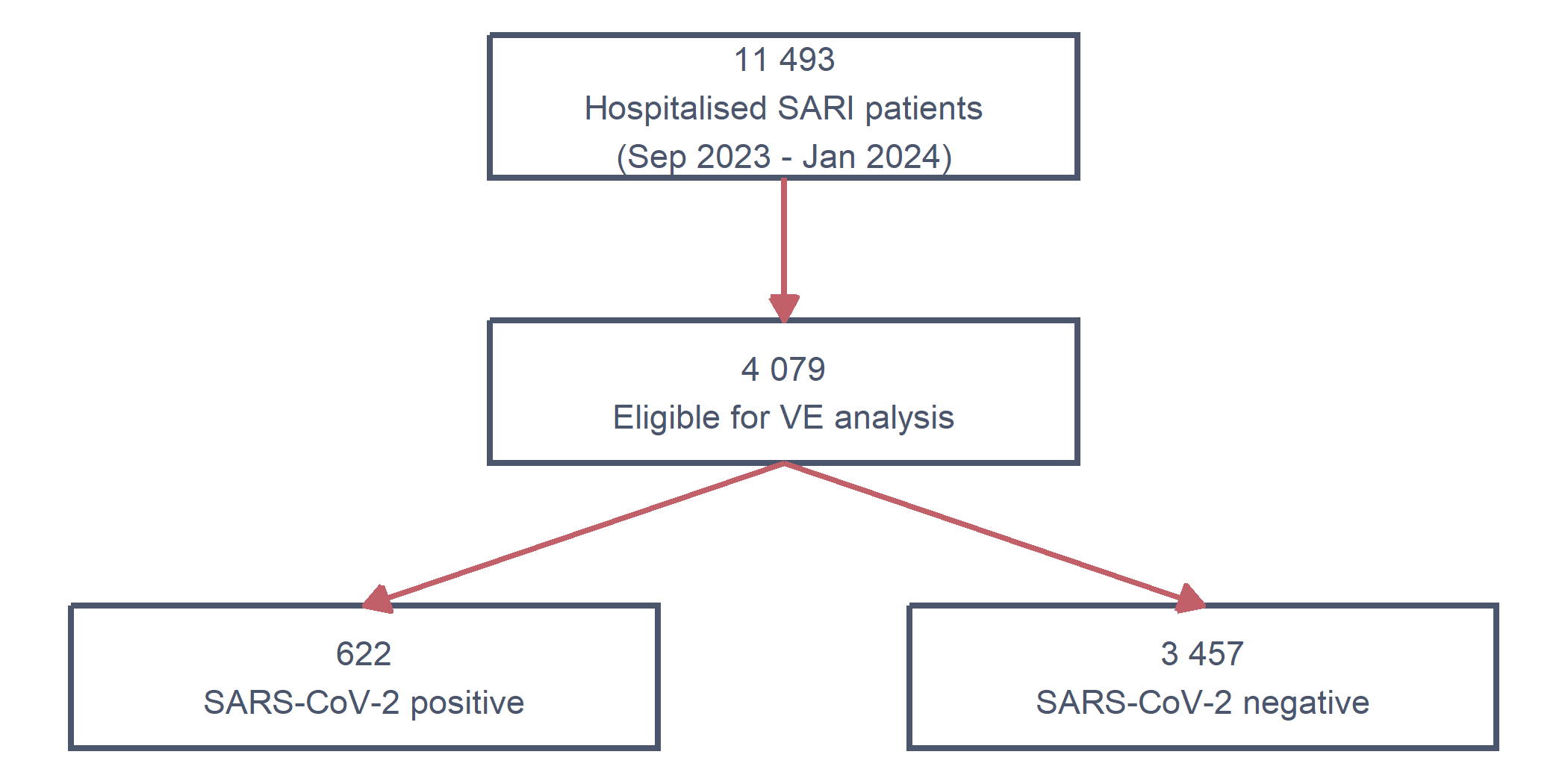


| **7 414 patients excluded** |
| --- |
| **2 243 outside the analysis period** |
| (1379 with date of symptom onset before the start of the campaign + 14 days, 459 from 1 site excluded due to vaccination data quality issues and 405 from 1 site with vaccination data issues before 13/11/2023) |
| **1 490 exclusion criteria (protocol)** |
| (19 missing consent information, 145 missing information on SARI case definition, 559 did not meet the ECDC SARI case definition and 767 had a missing RT-PCR test result/did not have an RT-PCR test) |
| **2 328 in an ineligible population group** |
| (12 healthcare workers, 403 residents in a long-term care facility, 1469 aged < 18 years and 444 outside the target group for vaccination) |
| **716 missing key covariates for analysis** |
| (4 missing swab date, 85 missing symptom onset date, 1 missing age, 3 missing sex, 176 missing information on common chronic diseases, 169 missing vaccination status and 278 missing date of last received vaccine dose) |
| **109 were ineligible due to timing of symptom onset, swab and hospitalisation** |
| (106 swabbed > 10 days after symptom onset and 3 swabbed > 3 days before symptom onset) |
| **229 with ineligible vaccination status** |
| (190 with last vaccine dose <14 days before symptom onset, 26 with last vaccine dose received within 6 months prior to the campaign, 12 vaccinated with bivalent vaccine and 1 received Comirnaty XBB.1.5 vaccine before 31/08/2023) |
| **202 records from 4 sites with less than 5 cases/controls or with no vaccinated patients** |
| **Records included are from 41 hospitals in 7 sites (Belgium, Spain, Croatia, Ireland, Lithuania, Malta and Navarre region (Spain))** |

## Supplementary Table S1. Start date of the 2023 autumn vaccination campaign by site, VEBIS hospital study, October 2023 - January 2024

| **Site** | **Start date vaccination campaign** |
| --- | --- |
| Belgium (BE) | 15 Sep 2023 |
| Croatia (HR) | 18 Sep 2023 |
| Czechia (CZ) | 04 Aug 2023 |
| Germany (DE) | 18 Sep 2023 |
| Hungary (HU) | 01 Oct 2023 |
| Ireland (IE) | 02 Oct 2023 |
| Lithuania (LT) | 05 Oct 2023 |
| Malta (MT) | 09 Oct 2023 |
| Navarre region, Spain (NA) | 16 Oct 2023^a^ |
| Portugal (PT) | 29 Sep 2023 |
| Romania (RO) | 02 Oct 2023 |
| Spain (ES) | 25 Sep 2023 |

1. The 2023 autumn vaccination campaign started earlier in the Navarre region in Spain (25 Sep 2023), with sparse administration of vaccines. Vaccines started being administered in larger quantities after 16 Oct 2023.

## Supplementary Table S2. Target groups for vaccination during the 2023 COVID-19 autumn vaccination campaign by site^a^, VEBIS hospital study, October 2023 - January 2024

| **Site** | **Priority groups based on age** | **Adults with underlying chronic conditions^b^** | **Pregnant women** |
| --- | --- | --- | --- |
| Belgium (BE) | ≥ 65 | Asthma; Diabetes; Heart disease; Immunodeficiencies; Liver disease; Lung disease; Neuromuscular disorder; Obesity; Renal disease | Yes |
| Croatia (HR) | ≥ 65 | Diabetes; Heart disease; Immunodeficiencies; Liver disease; Lung disease; Renal disease | Yes |
| Czechia (CZ) | ≥ 50 | Diabetes; Heart disease; Hypertension; Immunodeficiencies; Liver disease; Lung disease; Obesity; Renal disease | Yes |
| Germany (DE) | ≥ 60 | Diabetes; Heart disease; Immunodeficiencies; Liver disease; Lung disease; Renal disease | No |
| Hungary (HU) | ≥ 60 | Asthma; Cancer; Diabetes; Heart disease; Hypertension; Immunodeficiencies; Liver disease; Lung disease; Obesity; Renal disease | Yes |
| Ireland (IE) | ≥ 50 | Cancer; Diabetes; Heart disease; Immunodeficiencies; Liver disease; Lung disease; Neuromuscular disorder; Obesity; Renal disease | Yes |
| Lithuania (LT) | ≥ 65 | Cancer; Diabetes; Heart disease; Immunodeficiencies; Lung disease; Renal disease | Yes |
| Malta (MT) | ≥ 55 | Diabetes; Heart disease; Immunodeficiencies; Liver disease; Lung disease; Renal disease | Yes |
| Navarre region, Spain (NA) | ≥ 60 | Asthma; Diabetes; Heart disease; Immunodeficiencies; Liver disease; Lung disease; Obesity; Renal disease | Yes |
| Portugal (PT) | ≥ 60 | Asthma; Cancer; Diabetes; Heart disease; Hypertension; Immunodeficiencies; Lung disease; Obesity; Renal disease; Rheumatologic disease | Yes |
| Romania (RO) | ≥ 18 | Cancer; Diabetes; Heart disease; Immunodeficiencies; Liver disease; Lung disease; Neuromuscular disorder; Obesity; Renal disease | No |
| Spain (ES) | ≥ 60 | Asthma; Diabetes; Heart disease; Immunodeficiencies; Liver disease; Lung disease; Obesity; Renal disease | Yes |

^a^ Other groups, such as healthcare workers, persons living in long-term care facilities or nursing homes, children, etc, were not included in this table since they were not part of the study, although these groups might have been targeted for vaccination in their country.

^b^ Chronic conditions used in the study to identify patients targeted for vaccination in each site, based on the data collected. Other chronic conditions might have been part of the vaccination target in each site in addition to the ones presented on the table.
